# Supplementary material for: Hydrogen embrittlement in metallic nanowires
Source: Nat Commun. 2019 May 1;10:2004. doi: 10.1038/s41467-019-10035-0 (PMC6494841; doi:10.1038/s41467-019-10035-0)
Supplement: Supplementary file 1 — Supplementary Information [file 41467_2019_10035_MOESM1_ESM.docx]

**Supporting Information**

**Hydrogen embrittlement in metallic nanowires**

Sheng Yin^1*^, Guangming Cheng^2*^, Tzu-Hsuan Chang^2^, Gunther Richter^3^, Yong Zhu^2#^ and Huajian Gao^1#^

^1^School of Engineering, Brown University, Providence, RI 02912, USA

^2^Department of Mechanical and Aerospace Engineering, North Carolina State University, Raleigh, NC 27695, USA

^3^Max Planck Institute for Intelligent Systems, Heisenbergstrasse 3, D-70589 Stuttgart, Germany

^*^ These authors contributed equally to this work.

^#^ These authors jointly supervised this work.

#e-mail: yong_zhu@ncsu.edu; huajian_gao@brown.edu

**Supporting information includes:**

Supplementary Notes 1-9:

1. Characterization of Hydrogen diffusion in Ag NWs

2. DFT calculation of adsorption energy of Hydrogen on (001) Ag surface

3. Additional information on in situ TEM tensile tests

4. MD simulation: strain rate and temperature effects

5. Stress relaxation simulation in a penta-twinned NW with surface notch

6. Hydrogen effect zone in the surface ledge model

7. Activation energy change associated with successive dislocation nucleation

8. Localization via a nonhomogeneous Poisson process

9. Localization due to variance of hydrogen in the surface ledge model

**Supplementary Note 1.** **Characterization of Hydrogen diffusion in Ag NWs**

For hydrogen charging, a thin layer of Ag NWs (with a thickness of 0.5 mm) is dispersed on a Si substrate. The Ag NWs were from the same synthesis batch as the tested NWs in order to ensure the same crystalline quality and dimensions. The dispersed Ag NWs were randomly oriented forming a dense yet hydrogen permeable network^1^. Before hydrogen charging, the substrate containing Ag NWs was purged for 2 hrs in a vacuum chamber with a constant flow (~0.1 sccm per hr) of pure Ar (99.99999%) at 250 °C. Next, they were immersed into a constant flow (~0.1 sccm per hr) of H_2_/Ar (molar ratio, 1:1) for 12, 24, 36, 48, 60 and 72 hrs, at room temperature. Time-of-Flight Secondary Ion Mass Spectrometry (SIMS) was used to examine the relative concentration of hydrogen inside the samples with different charging time, shown in Supplementary Fig. 1. The relative hydrogen concentrations ($c_{H}$) in Ag NWs with different charging time were normalized by that in the as-received ones (not immersed in H_2_/Ar atmosphere), as shown in Supplementary Tab. 1. The hydrogen concentration in the samples increased with the increase of the charging time but saturated as the charging time reached 48 hrs.

Considering the high diffusivity of hydrogen in Ag (4.9x10^-12^ m^2^s^-1^) at room temperature and high formation energy of H interstitials, a high concentration of hydrogen should not remain in the bulk lattice of Ag after charging. But in our experiments, the NW networks showed a very high surface/volume ratio compared with the bulk counterpart. We attributed the time scale for the charging process and the high values of hydrogen concentration obtained from the SIMS experiments to the surface adsorption of Ag NWs during the charging process.

Hydrogen adsorption on metal surface involves two steps: dissociation of H_2_ molecule and transport of the chemisorbed hydrogen^2^. Flat and clean Ag surface is known to have a very high energy barrier for hydrogen dissociation and formation of an Ag-H adsorptive bond is endothermic^2-4^. Previous studies revealed that impurities atoms, such as surface and subsurface oxygen species, can promote dissociation of hydrogen molecules^5,6^. In the Ag NWs, the surface is not flat (e.g. roughness and surface steps/defects^7^). These conditions make hydrogen adsorption possible but the activation energy is still very high when compared with other FCC metals, like Cu or Pt^3,4^. The characteristic time scale of adsorption can be hours for this high activation energy of dissociation^6^, consistent with our experiments. As hydrogen can remain on or near the NW surface, the equilibrium concentration of hydrogen in our system can be high comparing with the bulk counterpart due to the large surface/volume ratio of NWs.

If we only consider monolayer hydrogen adsorption on Ag surface, the area of each possible adsorption site$s_{\text{H}}$on surface is $ka^{2}$, where *a* is the lattice constant and$k=0.2\sim1$is a coefficient depending on the surface orientation^8^. The volume per Ag atom in FCC lattice$v_{\text{Ag}}$equals a^3^/4. If we approximately treat the Ag NW cross section as a circle with radius of *r*, we can estimate the adsorption amount of hydrogen on the NW surface by:

$c_{\text{H}}=\frac{m_{\text{H}}}{m_{\text{Ag}}}\frac{N_{\text{H}}}{N_{\text{Ag}}}=\frac{m_{\text{H}}}{m_{\text{Ag}}}\frac{2\pi rL/s_{\text{H}}}{\pi r^{2}L/v_{\text{Ag}}}=\frac{m_{\text{H}}}{m_{\text{Ag}}}\frac{a}{2kr}$ (1)

where $m_{\text{H}}=\text{1.00794 u}$and $m_{\text{Ag}}=\text{107.8682 u}$are the atomic masses of hydrogen and silver atoms, respectively; $N_{\text{H}}$,$N_{\text{Ag}}$ are the total numbers of hydrogen and silver atoms, respectively; $L$ is the NW length; $a=0.408nm$ is the lattice constant of silver;$s_{\text{H}}=ka^{2}$is the surface area per adsorption site and$v_{\text{Ag}}=a^{3}/4$ is the volume per Ag atom.

For a NW with diameter of 70 nm ($r=35nm$), we can estimate the hydrogen concentration to be around 54-272 wt ppm, which is comparable to our experimental data. In our experimental conditions, the maximum hydrogen atoms absorbed on the NW surface (for a NW diameter of 70nm) is 8 atoms nm^-2^ (Supplementary Tab. 1), about 2/3 (surface coverage, $\theta_{H}$) of the (001) Ag surface (maximum around 12 atoms/nm^2^ on (100) surface).

**Supplementary Figure 1 | Comparison of H and Ag intensities in Ag NWs with different charging time into a H_2_/Ar (molar ratio, 1:1) atmosphere by using SIMS.**

**Supplementary Table 1 | Hydrogen difference (**$c_{\text{H}}$**) in Ag NWs with different charging time into a H_2_/Ar (molar ratio, 1:1) atmosphere, normalized to the as-received samples (not immersed in H_2_/Ar atmosphere).** The corresponding hydrogen atoms absorbed on the NW surface and surface coverage $\theta_{H}$ (for a NW diameter of 70 nm) were also given.

| Charging time | $\boldsymbol{c}_{\mathbf{H}}$ | | Surface adsorption | |
| --- | --- | --- | --- | --- |
| hrs | at. % | wt ppm | H atoms nm^-2^ | surface coverage $\theta_{H}$ |
| 0 | 0 | 0 | 0 | 0 |
| 12 | 0.15 | 14 | 2 | 0.17 |
| 24 | 0.41 | 38 | 4 | 0.33 |
| 36 | 0.63 | 59 | 7 | 0.58 |
| 48 | 0.8 | 75 | 8 | 0.67 |
| 60 | 0.8 | 75 | 8 | 0.67 |
| 72 | 0.81 | 76 | 8 | 0.67 |

**Supplementary Note 2. DFT calculation of adsorption energy of Hydrogen on (001) Ag surface**

To compute the adsorption energy of hydrogen on Ag surface, we use the DFT method implemented in the Vienna ab initio simulation package (VASP)^1^. The pseudopotentials used are of the PAW^2^ type and the exchange correlation energy is evaluated using the Perdew-Burke-Ernzerhof^3^ generalized gradient approximation (GGA). The electronic wave functions were represented in a plane-wave basis set with energy cut-off of 400 eV. 6x6x1 Monkhorst Pack integration scheme in k space were used. Residual forces after relaxation are smaller than 0.01 eVA^-1^. Dipole-dipole interactions are corrected for the calculation of energies.

The (001) Ag surface was considered for hydrogen adsorption. A slab of Ag was created with 8 atomic layers in the thickness direction. A 16 Å thick vacuum layer was put along the z-direction to mimic the free surface in the simulation cell. The cell volume and the two very bottom atomic layers were fixed during the calculations.

Adsorption energies were computed by subtracting the energies of the adsorbate atoms and the slab from the energy of the adsorbates/slab system as shown in the following equation:

$n\Delta E_{\mathrm{ad}}=E_{\mathrm{Ag}_{(001)}H_{n,ad}}-E_{\mathrm{Ag}_{\left( 001 \right)}}-E_{H_{n}}$ (2)

where $E_{\mathrm{Ag}_{(001)}H_{n,ads}}$ is the total energy of the system with adsorbed $H_{n}$, $E_{\mathrm{Ag}_{\left( 001 \right)}}$ is the energy of the Ag (001) slab, and $E_{H_{n}}$ is the energy of the adsorbates. With this definition, a negative $\Delta E_{\mathrm{ad}}$ indicates stable adsorption on the slab.

The adsorption energies of an H atom were calculated for the high-symmetry sites on the Ag surfaces, including top, bridge and four-fold hollow positions shown in Supplementary Fig. 2. The adsorption energies on the high-symmetry sites close to a surface ledge are also considered, as shown in Supplementary Fig. 3.


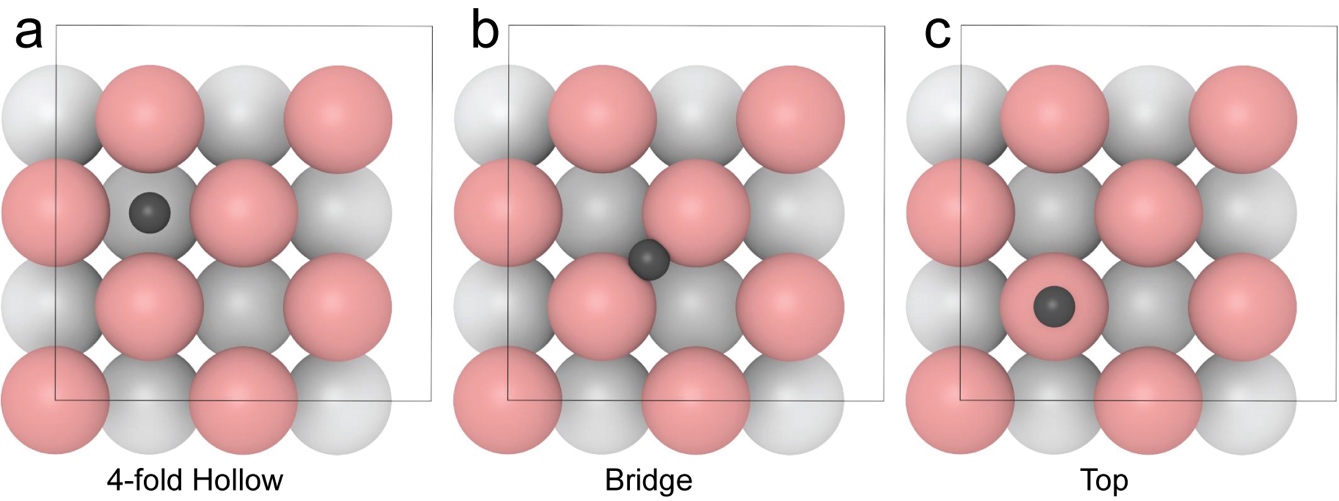


**Supplementary Figure 2 |** **High-symmetry sites for adsorption of an H atom on Ag (001) surface (top view).** **a,** 4-fold Hollow site. **b,** Bridge site. **c,** Top site. Pink and white spheres represent first and second layer atoms, respectively.


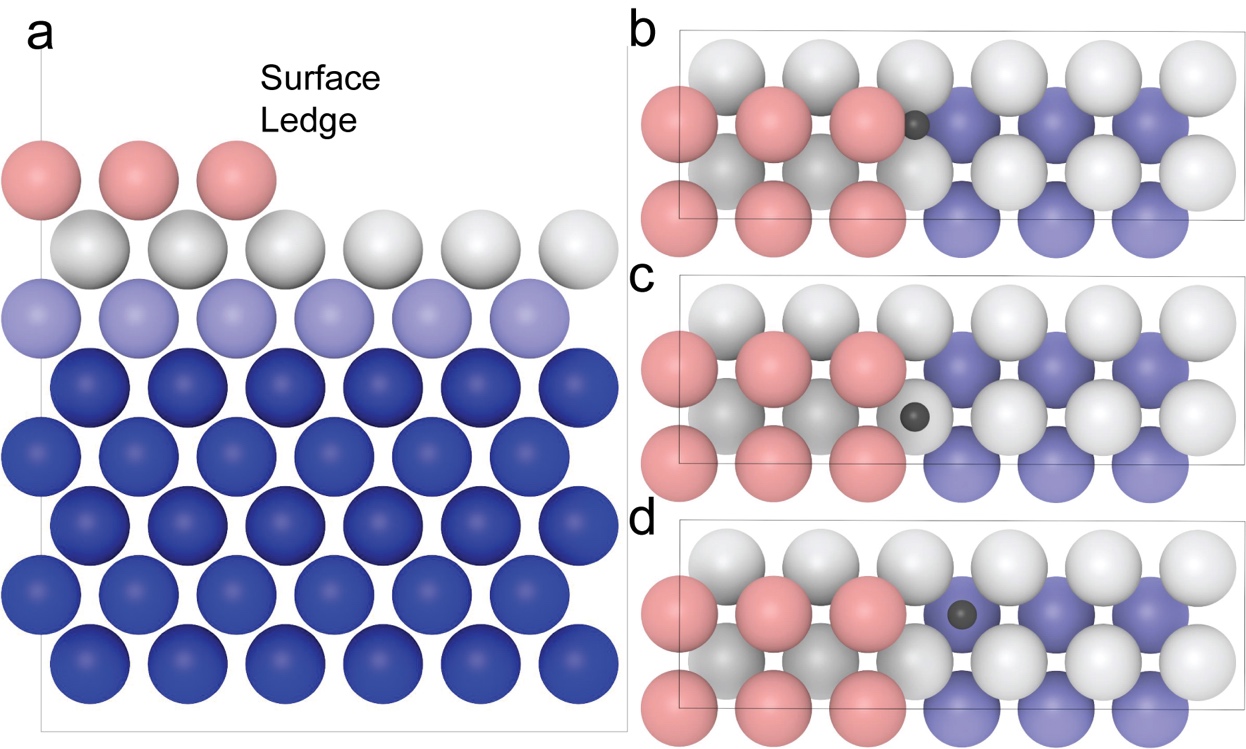


**Supplementary Figure 3 |** **High-symmetry sites for adsorption of an H atom on Ag (001) surface close to a surface ledge.** **a,** Side view of the atomistic model. The atoms are colored by the relative position in the height direction. **b-d,** Top view of the model. **b,** Bridge site. **c,** Top site. **d,** 4-fold Hollow site.

**Supplementary Table 2 | Adsorption energy of hydrogen adsorbate on (001) Ag surface.**

|  | Adsorption site | Adsorption Energy (eV) |
| --- | --- | --- |
|  | Top | -2.097 |
| On (001) Ag surface | Bridge | -3.030 |
|  | 4-fold Hollow | -3.023 |
| Below (001) Ag surface | Octahedral interstitial site | -2.549 |
|  | Top | -3.165 |
| Above (001) Ag surface with ledge | Bridge | -3.032 |
|  | 4-fold Hollow | -3.017 |

The calculated adsorption energies of hydrogen adsorbate on (001) Ag surface are shown in Supplementary Tab. 2. Although the adsorption barrier is high when considering the bond energy of the H_2_ molecule, the calculated adsorption energies at all the high-symmetry sites are negative, indicating stable adsorption on the metal surface. In addition, when a ledge exists on the free surface, as shown in Supplementary Fig. 3, the lowest adsorption energy further drops to -3.165 eV on the top site as shown in Supplementary Tab. 2. The interstitial energy of hydrogen atom was also calculated and included in Supplementary Tab. 2. The system has lower energy when hydrogen is adsorbed on bridge or 4-fold hollow surface sites than in an octahedral interstitial site. Previous studies indicate that impurity atoms, such as surface and subsurface oxygen species, can further decrease the adsorption energy^4, 5^. The DFT calculations confirm stable adsorption of hydrogen adsorbate on (001) Ag surface.

**Supplementary Note 3.** **Additional information on *in situ* TEM tensile tests**

The mechanical testing was carried out *in* *situ* inside a TEM using a MEMS-based material testing system, which consists of an electrostatic (comb-drive) actuator, a capacitive load sensor and a gap in between for mounting samples (Supplementary Fig. 4a). Displacement (and strain) is measured by digital image correlation of TEM images of the two deposited local markers on the specimen (Supplementary Fig. 4b). This MEMS-based system has a strain resolution of 0.01% (gage length 2 μm) and a stress resolution of 1.4 MPa (for example, for NW diameter of 104 nm).

**
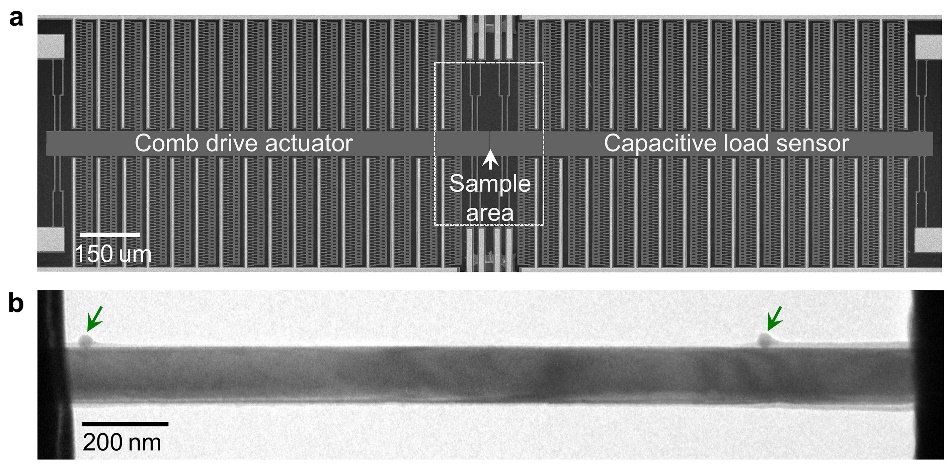
**

**Supplementary Figure 4 | A comb-drive-actuated MEMS testing system for *in situ* TEM tensile test.** **a,** Comb-drive-actuated MEMS device for tensile testing. **b,** TEM image showing a NW mounted on the device. Two local markers are deposited on the NW surface for displacement (or strain) measurement.


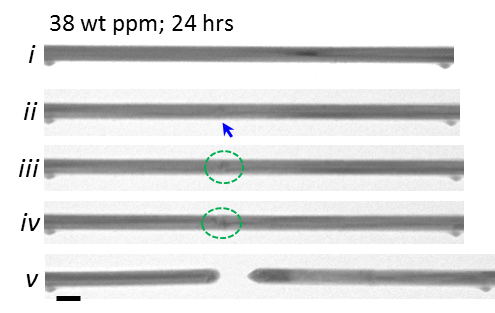


**Supplementary Figure 5 | Limited plasticity in a penta-twinned NW in the presence of hydrogen** ($c_{H}$ = 38 wt ppm, corresponding to the one in Fig. 1b). The nucleation and propagation of dislocations were marked by green arrows and ovals, respectively. i to iv correspond to the strains of 0, 1.5, 1.8 and 2.7%. Scale bar, 100 nm.


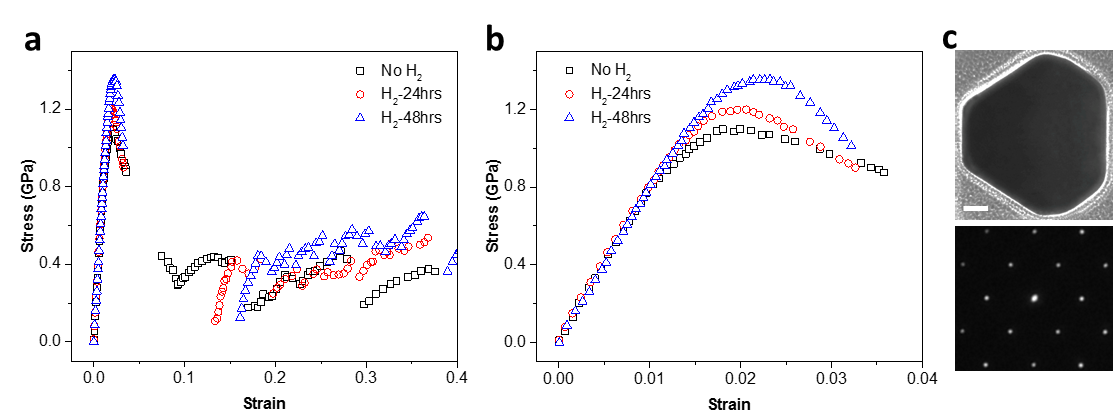


**Supplementary Figure 6 |** **Stress-strain responses (a,b) for single crystalline Ag NWs at different H concentrations.** **b** shows the details of stress-strain curves at a magnified strain scale from 0 to 0.04 as shown in **a**. The strain rate for in situ TEM tensile testing was ~0.005%/s. **c**, Cross-sectional TEM image of a single crystalline Ag NW and the corresponding diffraction pattern taken from <110> zone axis. Scale bar, 20 nm.

**Supplementary Note 4. MD simulation: strain rate and temperature effects**

To capture the hydrogen effect in simulations, the loading time scale should be comparable to the diffusion time scale of hydrogen, otherwise hydrogen would behave like sessile inclusions and promote dislocation nucleation as a result of lattice distortion, thus the strain rate and temperature is of critical importance in current simulations. As showed in Supplementary Fig. 7, tensile simulations at a fixed strain rate of 10^6^ s^-1^ were carried out at different temperatures, range from 100K to 800K. At higher temperature, such as 600K and 800K, the hydrogen atoms have enough time to diffuse and delay the surface nucleation. When temperature decreases to 300K, the nucleation strains for both cases are comparable. However, when temperature keeps decreasing to 100K, the sessile hydrogen will promote dislocation nucleation. Increasing the strain rate has similar effect as decreasing temperature. In Supplementary Fig. 8, we investigated the 300K case at higher strain rate of 10^8^ s^-1^. Comparing with Supplementary Fig. 7b, the difference is obvious. The presence of hydrogen promotes dislocation nucleation in this case due to the limited hydrogen diffusion at higher strain rate. These results further stress that the diffusion and interaction time scale of hydrogen is of critical importance in the simulations of surface nucleation.


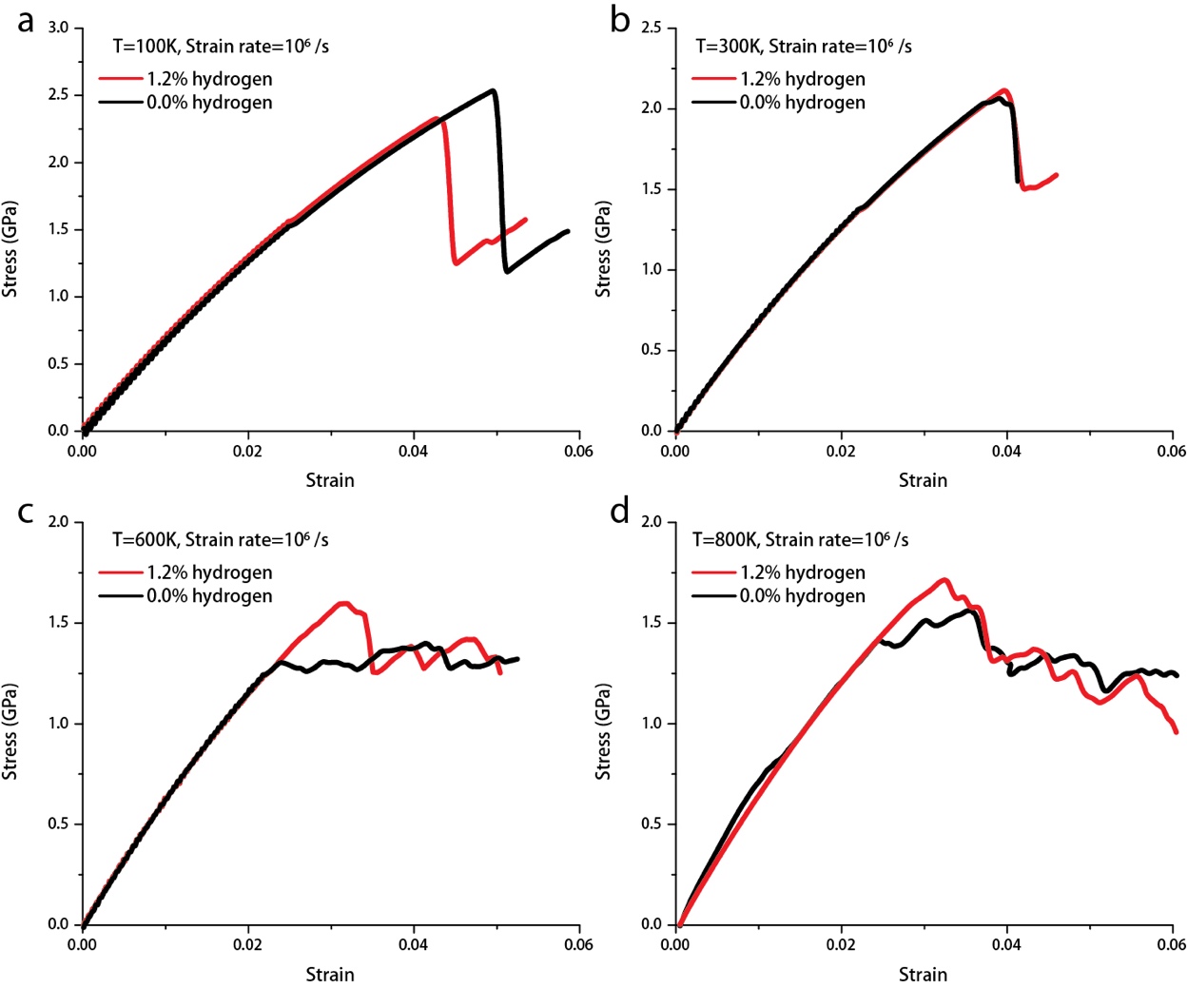


**Supplementary Figure 7 | Strain rate and temperature effects in MD simulations. a-d,** Temperature ranges from 100K to 800K at a fixed strain rate of 10^6^ s^-1^. Lower strain rate and higher temperature facilitate hydrogen diffusion in the simulation scale and allow hydrogen atoms to suppress surface dislocation nucleation.


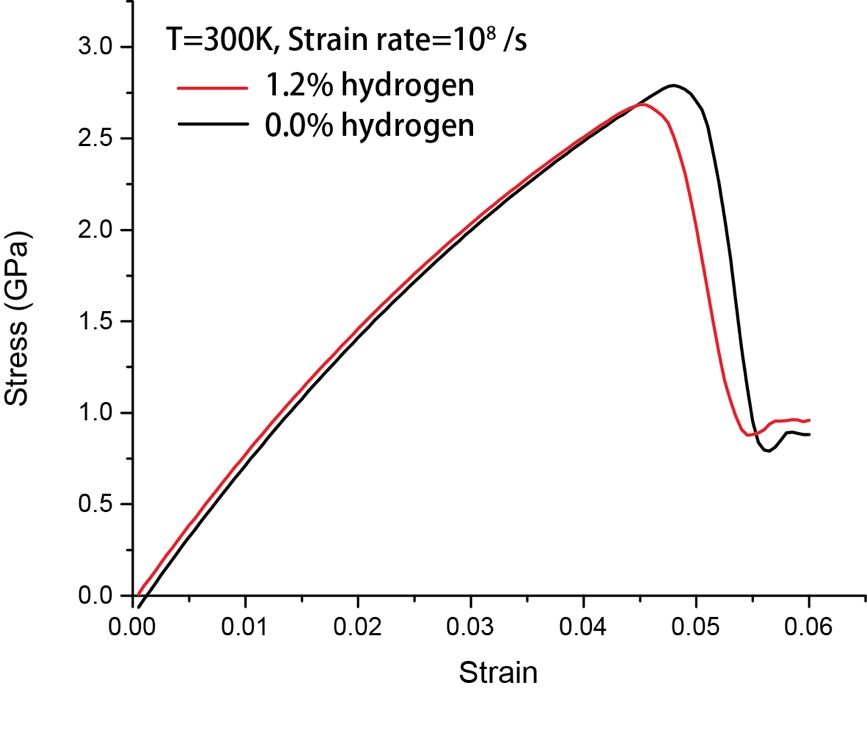


**Supplementary Figure 8 | Strain rate and temperature effects in MD simulations.** Temperature is 300K and strain rate is 10^8^ s^-1^. The nucleation occurred earlier with the presence of hydrogen at higher strain rate of 10^8^ s^-1^ comparing with Supplementary Fig. 7b.


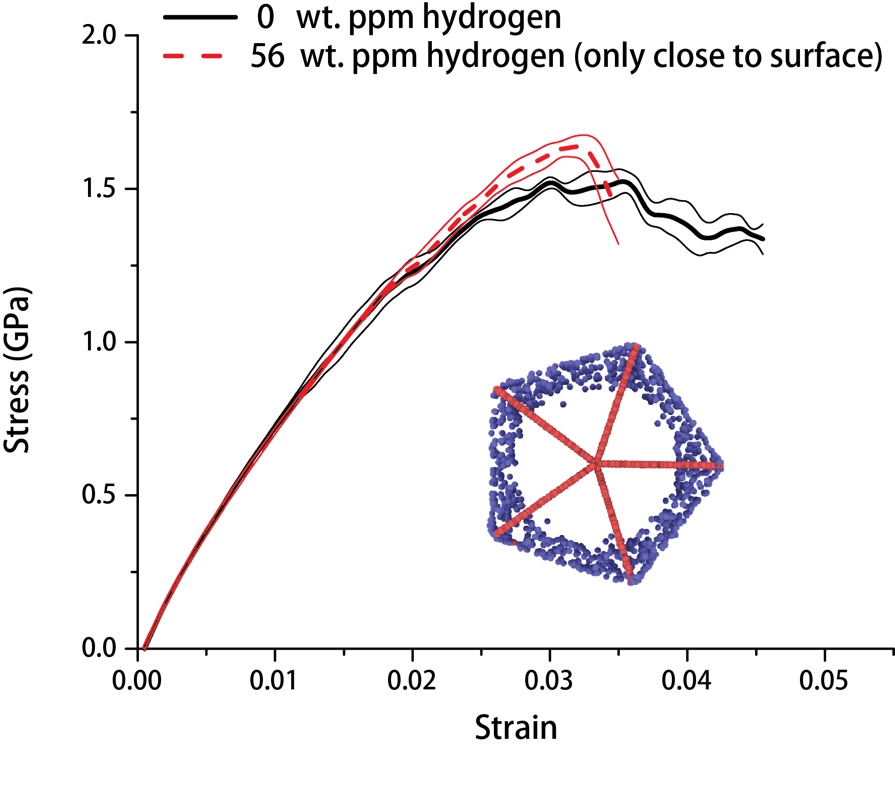


**Supplementary Figure 9 | Stress-strain curves with hydrogen close to the NW surface.** Blue atoms in the inserted figure are hydrogen atoms. With the hydrogen atom only charged on several surface layers, they can still effectively delay surface dislocation nucleation.

**Supplementary Note 5. Stress relaxation simulation in a penta-twinned NW with surface notch**


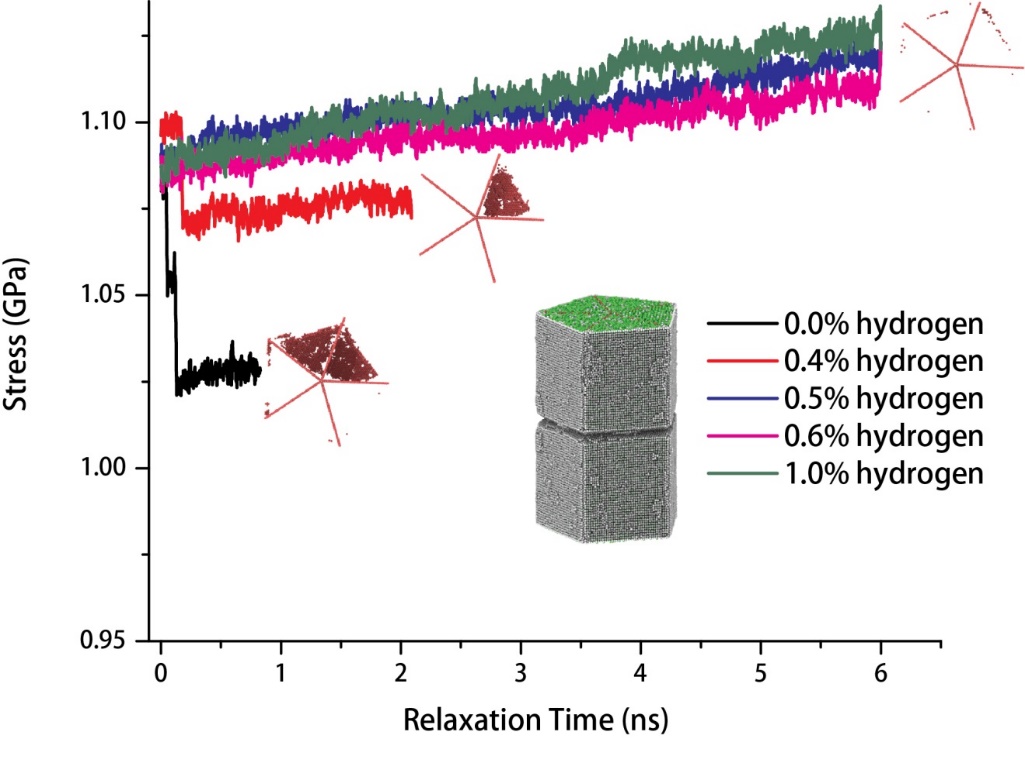


**Supplementary Figure 10 | Stress relaxation simulations of a penta-twinned NW with surface notch.** Hydrogen atoms accumulated around surface notch and suppress dislocation nucleation from notch tip.

**Supplementary Note 6. Hydrogen effect on stress distribution near a surface ledge**

**
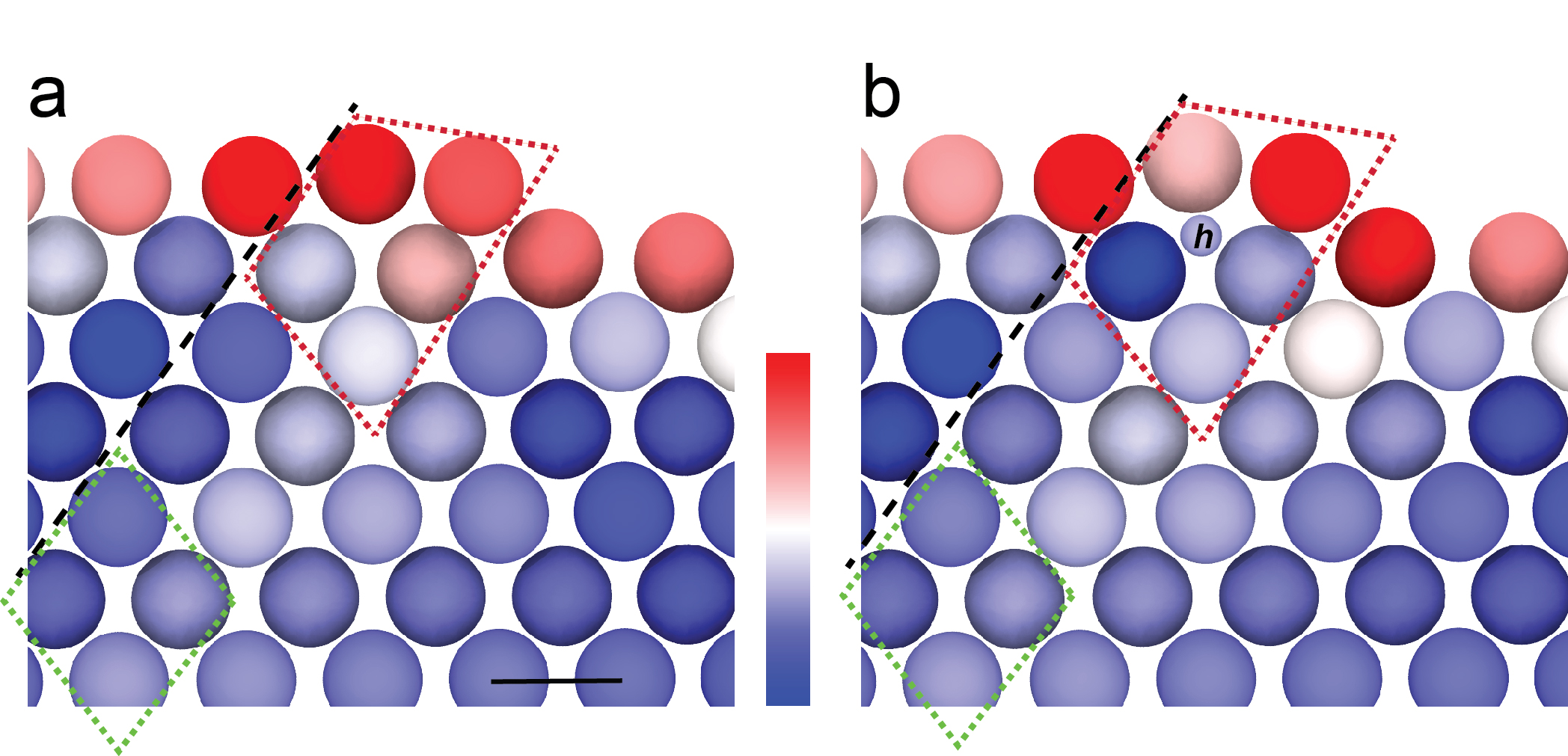
**

**Supplementary Figure 11 | Atomic von Mises stress distribution around a surface ledge** (**a**) without and (**b**) with hydrogen atoms in the interstitial sites next to the ledge. Scale bar, 0.3 nm. **a-b,** show the atomic von Mises stress distribution of a surface ledge under 4.5% tensile strain. The black dashed line shows the slip plane. With the existence of a hydrogen atom, the average von Mises stress around the surface ledge (red dashed region) in **b** is 25% lower than that without hydrogen in **a**. The hydrogen effect decays rapidly with distance, with only 2.5% of difference in average stress in the dashed green region.


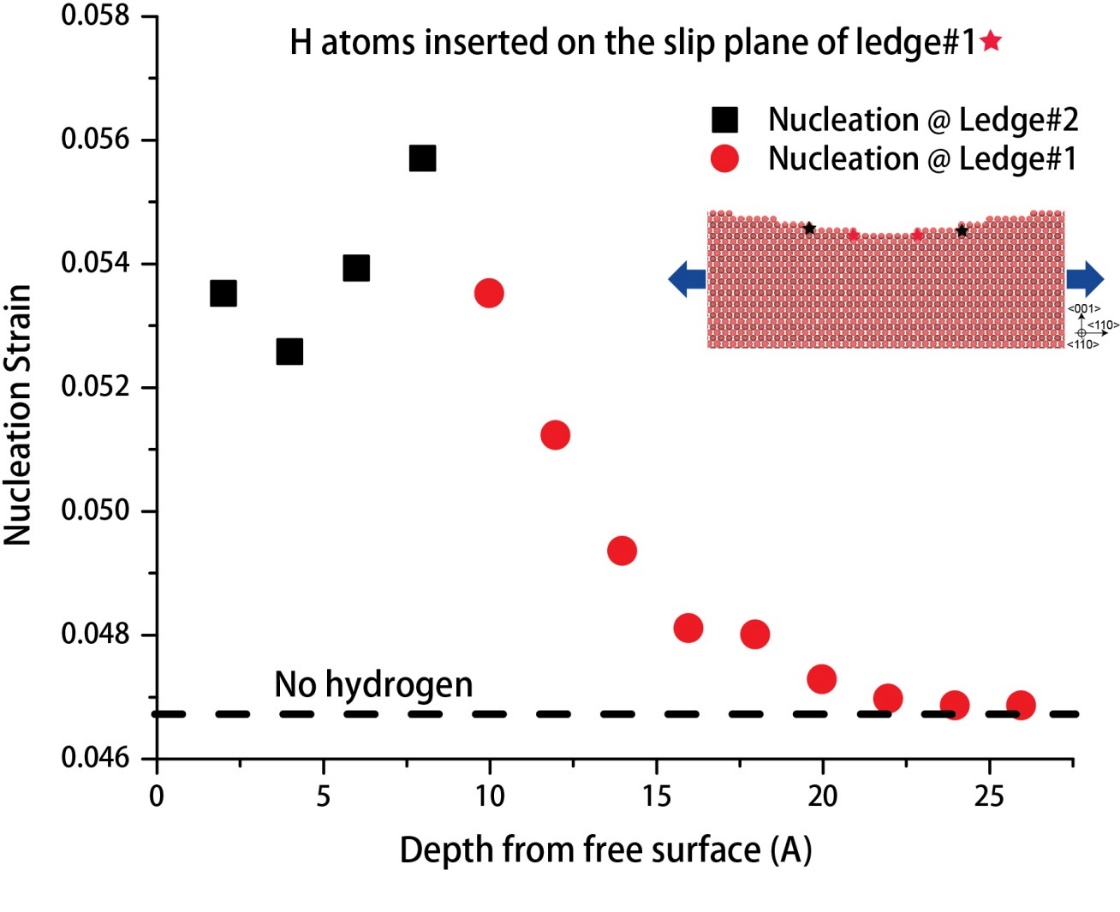


**Supplementary Figure 12 | Hydrogen effect zone from the surface ledge.** Ledge#1 and #2 are noted as red and black stars in the inset. Two rows of hydrogen atoms were inserted in the interstitial sites on the slip plane of ledge#1 at different depths from the free surface. Within 1nm from the free surface, the nucleation is suppressed by hydrogen atoms, initial dislocation nucleation occurred at ledge#2. As the distance between hydrogen and ledge#1 increases, hydrogen’s suppression effect keeps decreasing, and initial nucleation site will change to ledge#1. Finally, the suppression effect vanishes when hydrogen atoms are over 2.5 nm from the surface ledge.


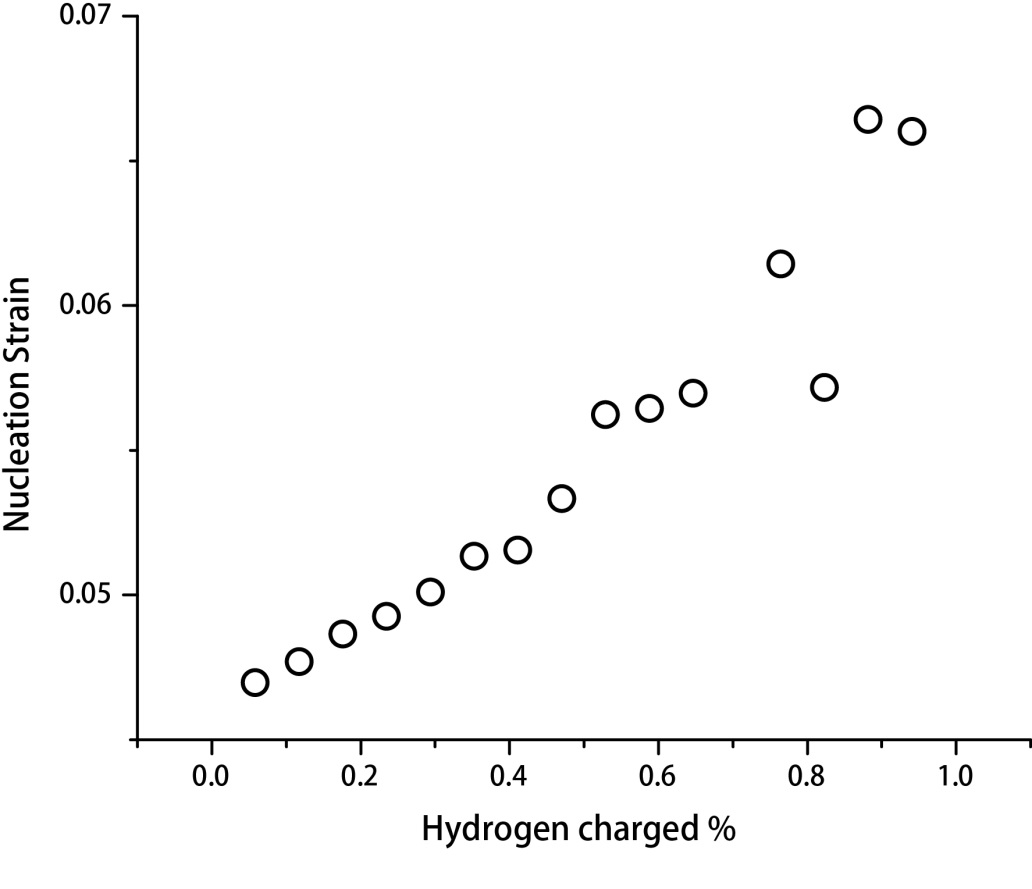


**Supplementary Figure 13 | Hydrogen atoms’ suppression effect when partially occupied the surface ledge sites.** Ideally all the interstitial sites at the surface ledge in the out of plane direction were charged with hydrogen atoms. When we keep decreasing the hydrogen density (still keep hydrogen evenly distributed), the suppression effect of hydrogen keeps decreasing, but it still can effectively suppress dislocation nucleation even if only half of interstitial sites were occupied by hydrogen.

**Supplementary Note 7. Activation energy change associated with successive dislocation nucleation**


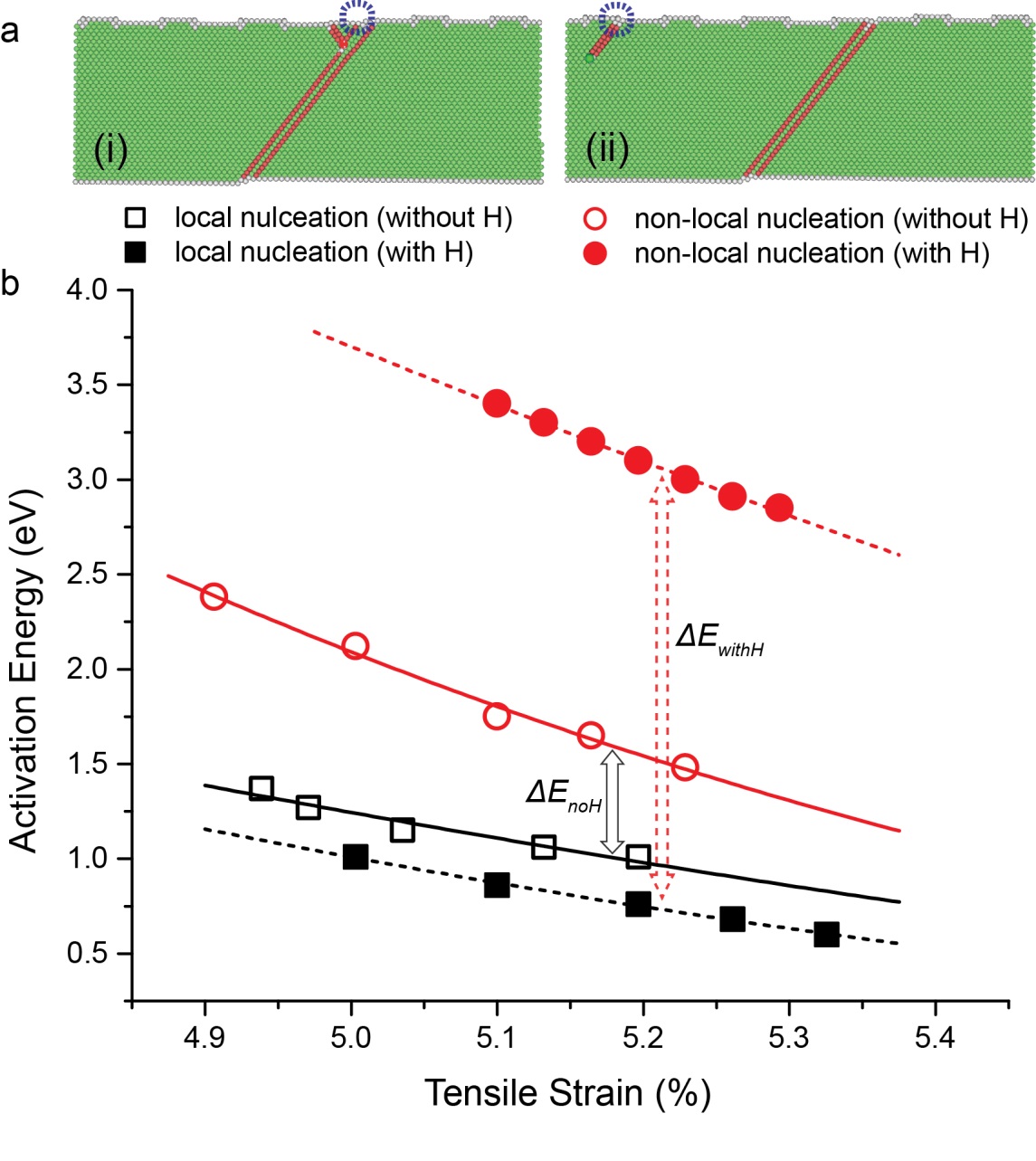


**Supplementary Figure 14 | NEB calculation of the nucleation activation energy change. a,** Two different nucleation configurations in the surface ledge model. (i), local nucleation: new partial nucleated at the extrinsic stacking fault created by the initial nucleation (blue dashed circle). (ii), non-local nucleation: new partial nucleated (blue dashed circle) at a surface ledge far away from the initial nucleation site where the extrinsic stacking fault was left. **b,** Activation energies of two different scenarios with/without hydrogen as functions of the tensile strain.

To explore the local activation energy change associated with succeeding dislocation nucleation, a 2d surface ledge model with twelve equivalent surface ledges was created. The simulation box was oriented along x-[110], y-[001], z-[$\bar{1}10$], with dimensions of 26 nmx50 nmx10 nm. Periodic boundary conditions were applied in the x and z direction. Twelve equivalent surface ledges were created on the (001) surface, which act as preferred surface nucleation sites. NEB method was applied to this ledge model. After initial nucleation, usually an extrinsic stacking fault will be left in the sample. The activation energies of succeeding nucleation events, either directly at the initial nucleation site (local nucleation, Supplementary Fig. 14a-i) or from a ledge away from the initial nucleation site (non-local nucleation, Supplementary Fig. 14a-ii) were calculated. Nucleation with and without hydrogen at the selected nucleation ledges were considered. For comparison, the activation energies were plotted as a function of tensile stain in Supplementary Fig. 14b. Two solid lines represent local nucleation and non-local nucleation without hydrogen. Two dashed lines represent local nucleation and non-local nucleation with hydrogen at the specific nucleation ledge. The NEB results show that in the current model, the activation energy of local nucleation is lower than that of non-local nucleation. The activation energy of local nucleation decreases in the presence of hydrogen, while the activation energy of non-local nucleation increases significantly due to the effect of hydrogen. Overall, the presence of hydrogen substantially increases $\Delta E$ between non-local nucleation and local nucleation to promote localized plasticity, as shown in Supplementary Fig. 14b.

**Supplementary Note 8. Localization via a nonhomogeneous Poisson process**

Notations:

- Vector of dislocation nucleation sites $S=\{s_{1},s_{2},...,s_{N}\}$. We assume the sites to be exchangeable.
- Event of interest: dislocation nucleation E. We assume E could only occur once at each nucleation site, potential successive dislocation nucleation at the same site as described in S6 is not included in this model and can be considered to occur after time interval$\text{δt}$. Furthermore, once E has occurred for any other site or any multiple sites simultaneously (that is, within a short time interval$\text{δt}$), the process shall be terminated.
- Random variable: dislocation nucleation activation energy Q. At any instance t, the rate at which E occurs (denote as$\lambda$) equals:

$\lambda=v_{0}\exp\{-\frac{Q}{kT}\}$ (3)

where$v_{0}$,$k$and$T$are constants independent of site s or time t.

- Random variable time to event$\tau$, for each site s.

How Q and$\lambda$change through time:

- When t=0, $Q(s,t)=Q_{0}$
- When t>0

$Q(s,t)\sim Normal\text{ }(\bar{Q}(t),\sigma_{Q}(t))$ (4)

where $\bar{Q}(t)$ is deterministically determined by the loading process, while variance $\sigma_{Q}(t)$ is assumed to be:

$\sigma_{Q}(t)=r\bar{Q}(t)$ (5)

Importantly, we assume r to be constant through time. Therefore, random variable$-\frac{Q(s,t)}{kT}$follows the normal distribution as well:

(6)

- Consequently$\lambda(s,t)$follows log normal distribution.

Localization:

- To model localization, we consider the following scenario: suppose E first occurs at some site at time$\tau_{1}$. On one hand, if E would not occur at any other site between$\tau_{1}$and$\tau_{1}+\text{δt}$, the process would be terminated, and we call this localization. On the other hand, if E occurs at another site between time$\tau_{1}$and $\tau_{1}+\text{δt}$ (call them$\tau_{2}$,$\tau_{3}$, … etc.), we think of this as simultaneous distributed events.
- We model the occurrence of E for each site as a (non-homogeneous) Poisson process, governed by the changing rate $\lambda(s,t)$. Consider some arbitrary time point$\tau_{0}$, then the probability that $\tau_{1}>\tau_{0}$equals:

$P(\tau_{1}>\tau_{0})=\prod_{S} P(\tau(s)>\tau_{0})=\prod_{S} e^{-m(s,\tau_{0})}$ (7)

where

$m(s,\tau_{0})=\int_{0}^{\tau_{0}} \lambda(s,u)du$ (8)

We could use this formula to combine the Poisson process across all nucleation sites. (Important note: this is a nice feature when modeling independent Poisson process. In reality, once E occurs at some site, we should no longer view Poisson processes for different sites as independent. However, we make this simplification here for mathematical simplicity, and we argue that within very short time interval$\text{δt}$after$\tau_{1}$, it’s reasonable to still assume independence).

$P(\tau_{1}>\tau_{0})=\prod_{S} e^{-m(s,\tau_{0})}=\exp\{-\sum_{S} m(s,\tau_{0})\}$ (9)

where

$\sum_{S} m(s,\tau_{0})=\sum_{S} \int_{0}^{\tau_{0}} \lambda(s,u)du=\int_{0}^{\tau_{0}} \sum_{S} \lambda(s,u)du$ (10)

In other words, we model a “combined” Poisson process with rate at time t as$\lambda_{s}(t)=\sum_{s} \lambda(s,t)$. Again, each $\lambda(s,t)$ follows a log normal distribution.

- Now we calculate the probability that, conditioned on$\tau_{1}>\tau_{0}$, the first E still hasn’t occurred by $\tau_{0}+\text{δt}$:

$P(\tau_{1}>\tau_{0}+\text{δt}|\tau_{1}>\tau_{0})=\exp\{-m_{S}(\tau_{0},\tau_{0}+\text{δt})\}$ (11)

Where

$m_{S}(\tau_{0},\tau_{0}+\text{δt})=\int_{\tau_{0}}^{\tau_{0}+\text{δt}} \sum_{S} \lambda(s,u)du$ (12)

Furthermore, let’s assume$\text{δt}$is small enough that$\lambda(s,t)=\lambda(s,\tau_{0})$when$\tau_{0}<t<\tau_{0}+\text{δt}$, and thus$\int_{\tau_{0}}^{\tau_{0}+\text{δt}} \sum_{S} \lambda(s,u)du=\sum_{S} \lambda(s,\tau_{0})\text{δt}$.

Then the above probability equals:

$P(\tau_{1}>\tau_{0}+\text{δt}|\tau_{1}>\tau_{0})=\exp\{-\sum_{S} \lambda(s,\tau_{0})\text{δt}\}$ (13)

- Next, we calculate the probability that, conditional on $\tau_{1}>\tau_{0}$, the first E occurred before $\tau_{0}+\text{δt}$ while no other E occurred by$\tau_{0}+\text{δt}$. That is$\tau_{0}<\tau_{1}<\tau_{0}+\text{δt}$and$\tau_{2}>\tau_{0}+\text{δt}$ (i.e., E only occurs at one site, which is localization).

The probability that the first E occurring at a certain site$s'$and between$\tau_{0}$and$\tau_{0}+\text{δt}$ is:

$\lambda(s',\tau_{0})\text{δt}\exp\{-\lambda(s',\tau_{0})\text{δt}\}$ (14)

The probability that no other E occurred by$\tau_{0}+\text{δt}$is:

$\exp\{-m_{S/s'}(\tau_{0},\tau_{0}+\text{δt})\}=\exp\{-\sum_{S/s'} \lambda(s,\tau_{0})\text{δt}\}$ (15)

where$\sum_{S/s'} \lambda(s,\tau_{0})$means sum over all sites except for site $s'$.

The product of these two probabilities is

$\lambda(s',\tau_{0})\text{δt}\exp\{-\sum_{S} \lambda(s,\tau_{0})\text{δt}\}$ (16)

Since we only care about the upper bound on the probability of localization, we need to take maximum over$s'$:

$P_{\text{localization}}=(\max_{s'} \lambda(s',\tau_{0}))\text{δt}\exp\{-\sum_{S} \lambda(s,\tau_{0})\text{δt}\}$ (17)

The important term here is clearly $\lambda(s',\tau_{0})$

Since we know that

Consequently, $\lambda=v_{0}\exp\{-\frac{Q}{kT}\}$ follows the log normal distribution, it’s clear that $\max_{s'} \lambda(s',\tau_{0})$ would increase with bigger $\sigma_{Q}(t)$.

The hydrogen atoms increase the energy barrier for surface dislocation nucleation, increase the variance of the activation energy for different nucleation sites at the same time, and facilitate localization nucleation in penta-twinned NWs.

**Supplementary Note 9. Localization due to variance of hydrogen in the surface ledge model**

**
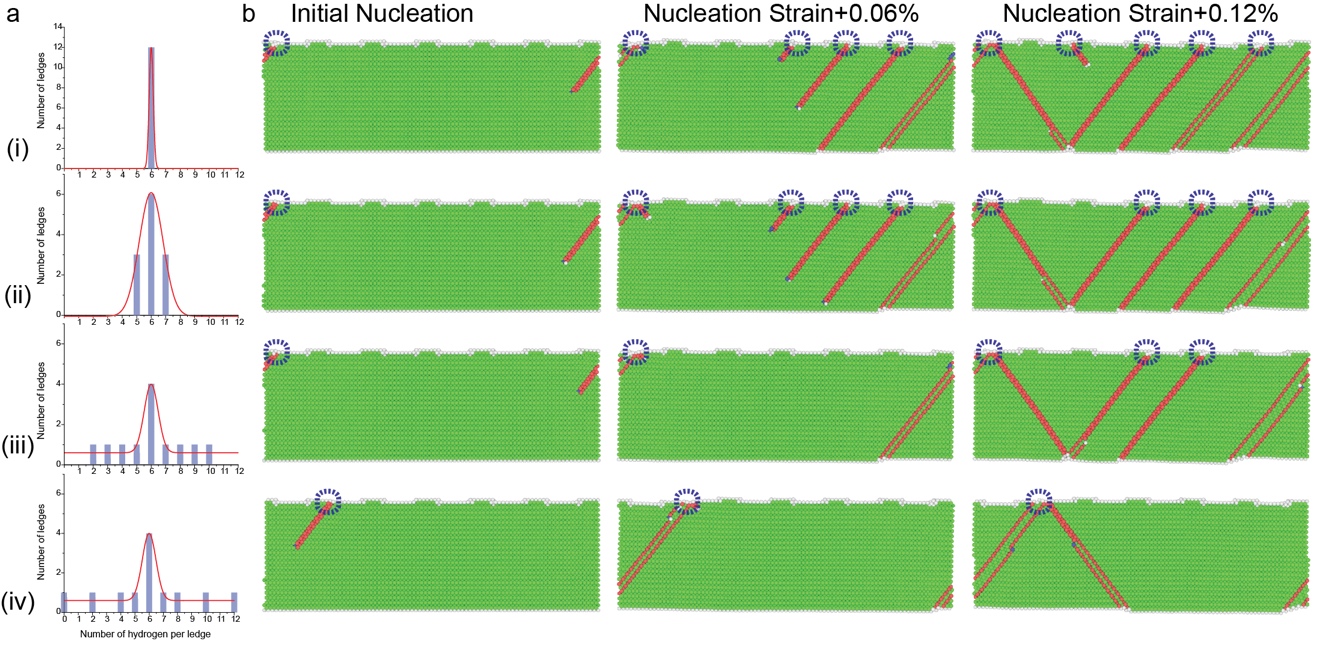
**

**Supplementary Figure 15 | Localization in the surface ledge model.** **a,** Hydrogen distribution in twelve surface ledges. The total number of hydrogen atoms are fixed, but the variance of concentration of hydrogen on surface ledges keeps increasing from (i)-(iv). **b,** Snapshots of nucleation with different distributions of hydrogen from initial nucleation to 12ps after initial nucleation (extra 0.12% strain). The nucleation sites were marked by blue circles.

To support the nonhomogeneous Poisson model in Supplementary Note 8 that increment in the variance of the activation energy for different nucleation sites could facilitate localization, surface ledge model simulations with different distributions of hydrogen were carried out. According to Supplementary Fig. 13, the increment of activation energy of surface ledge is directly correlated with hydrogen number charged per-ledge. The nonuniformity of activation energy was created through non-uniform hydrogen distribution on surface ledges. The simulation box was oriented along x-[110], y-[001], z-[$\bar{1}10$], with dimensions of 26 nmx50 nmx10 nm. Periodic boundary conditions were applied in the x and z directions. Twelve equivalent surface ledges were created on the (001) surface, which act as preferred surface nucleation sites. A constant number of hydrogen atoms were inserted in these ledges with different distributions, as shown in Supplementary Fig. 15a. The system was relaxed and equilibrated at temperature of 5K and then tensile loading was applied in the x direction at a strain rate of 10^8^/s. Surface dislocation nucleation was monitored and configurations of the system from initial nucleation to 12ps after initial nucleation (extra 0.12% strain) were shown in Supplementary Fig. 15b.

Hydrogen was distributed to the twelve surface ledges in a normal distribution with the same average concentration in cases (i)-(iv), as shown in Supplementary Fig. 15a. As the variance of hydrogen distribution increased, according to Supplementary Fig. 13, the variance of activation energy for different nucleation site was also enhanced. In case (i), all the 12 surface ledges contain the same number of hydrogen (same activation energy), and after initial nucleation, we can observe multiple nucleation on other sites as marked by blue circles. 5 different nucleation sites were observed within 12ps. When the variance of hydrogen distribution increased, as in (ii)-(iii), the number of activated nucleation sites decreased from 4 to 3. In case (iv), when the variance further increased, after initial nucleation, dislocation activities were localized to this initial weakened point, and only 1 nucleation was observed within 12ps.

The simulation results thus support our statistical analysis in Supplementary Note 8 and showed the importance of the variance in activation energy in transition from distributed plasticity to localized plasticity.

**Supplementary References**

1 Xu, F. & Zhu, Y. Highly conductive and stretchable silver nanowire conductors. *Adv Mater* **24**, 5117-5122 (2012).

2 Wandelt, K. *Surface and Interface Science: Solid Gas Interfaces II*. Vol. 6 (John Wiley & Sons, 2015).

3 Gomez, T., Florez, E., Rodriguez, J. A. & Illas, F. Reactivity of transition metals (Pd, Pt, Cu, Ag, Au) toward molecular hydrogen dissociation: extended surfaces versus particles supported on TiC (001) or small is not always better and large is not always bad. *The Journal of Physical Chemistry C* **115**, 11666-11672 (2011).

4 Greeley, J. & Mavrikakis, M. Surface and subsurface hydrogen: Adsorption properties on transition metals and near-surface alloys. *The Journal of Physical Chemistry B* **109**, 3460-3471 (2005).

5 Mohammad, A. B., Lim, K. H., Yudanov, I. V., Neyman, K. M. & Rösch, N. A computational study of H 2 dissociation on silver surfaces: The effect of oxygen in the added row structure of Ag (110). *Phys Chem Chem Phys* **9**, 1247-1254 (2007).

6 Xu, Y., Greeley, J. & Mavrikakis, M. Effect of subsurface oxygen on the reactivity of the Ag (111) surface. *J Am Chem Soc* **127**, 12823-12827 (2005).

7 Ramachandramoorthy, R. *et al.* Reliability of single crystal silver nanowire-based systems: stress assisted instabilities. *Acs Nano* **11**, 4768-4776 (2017).

8 Daw, M. S. & Baskes, M. I. Embedded-atom method: Derivation and application to impurities, surfaces, and other defects in metals. *Physical Review B* **29**, 6443 (1984).
