# Supplementary material for: Hydrogen embrittlement in metallic nanowires
Source: Nat Commun. 2019 May 1;10:2004. doi: 10.1038/s41467-019-10035-0 (PMC6494841; doi:10.1038/s41467-019-10035-0)
Supplement: Supplementary file 3 — Description of Additional Supplementary Files [file 41467_2019_10035_MOESM3_ESM.pdf]

Description of Additional Supplementary Files

File Name: Supplementary Movie 1
Description: Mechanical response and microstructure evolution of a pentatwinned Ag NW with absence of hydrogen. The NW shows large fracture strain (4.92%) owing to multiple necking.

File Name: Supplementary Movie 2
Description: Mechanical response and microstructure evolution of a pentatwinned Ag NW with presence of hydrogen (c_H = 75 wt ppm). Limited plasticity in the NW (an elongation of 2.32%) as a result of hydrogen embrittlement.
